# Anti-Neuroinflammatory Potential of Areca Nut Extract and Its Bioactive Compounds in Anthracene-Induced BV-2 Microglial Cell Activation

**DOI:** 10.3390/nu16172882

**Published:** 2024-08-28

**Authors:** Sakawrat Janpaijit, Monruedee Sukprasansap, Tewin Tencomnao, Anchalee Prasansuklab

**Affiliations:** 1 College of Public Health Sciences, Chulalongkorn University, Bangkok 10330, Thailand; sakawrat.j@chula.ac.th; 2 Center of Excellence on Natural Products for Neuroprotection and Anti-Ageing (Neur-Age Natura), Faculty of Allied Health Sciences, Chulalongkorn University, Bangkok 10330, Thailand; tewin.t@chula.ac.th; 3 Food Toxicology Unit, Institute of Nutrition, Mahidol University, Salaya Campus, Phutthamonthon, Nakhon Pathom 73170, Thailand; monruedee.suk@mahidol.edu; 4 Department of Clinical Chemistry, Faculty of Allied Health Sciences, Chulalongkorn University, Bangkok 10330, Thailand

**Keywords:** areca nut, (−)-epicatechin, arecoline, air pollutant, polycyclic aromatic hydrocarbon, microglial cell, neuroinflammation, NF-κB

## Abstract

Particulate matter (PM_2.5_) containing polycyclic aromatic hydrocarbons (PAHs) is of considerable environmental importance worldwide due to its adverse effects on human health, which are associated with neurodegenerative diseases (NDDs). *Areca catechu* L. (AC) fruit is known to possess various pharmacological properties; however, the anti-neuroinflammatory roles of AC on the suppression of PAH-induced neuroinflammation are still limited. Thus, we focused on the effects and related signaling cascades of AC and its active compounds against anthracene-induced toxicity and inflammation in mouse microglial BV-2 cells. Phytochemicals in the ethanolic extract of AC (ACEE) were identified using LC-MS, and molecular docking was conducted to screen the interaction between compounds and target proteins. Significant bioactive compounds in ACEE such as arecoline, (−)-epicatechin, and syringic acid were evinced through the LC-MS spectrum. The docking study revealed that (−)-epicatechin showed the highest binding affinities against NF-κB. For cell-based approaches, anthracene induced intracellular ROS, mRNA levels of TNF-α, IL-1β, and IL-6, and the release of TNF-α through enhancing JNK, p38, and NF-κB signaling pathways. However, the co-treatment of cells with ACEE or (−)-epicatechin could reverse those anthracene-induced changes. The overall study suggested that ACEE-derived bioactive compounds such as (−)-epicatechin may be developed as a potential anti-neuroinflammatory agent by preventing inflammation-mediated NDDs.

## 1. Introduction

Air pollution, which contains various substances such as biological components, particulate matter, and gases, is regarded as one of the significant environmental threats. It presents not only human health alterations but also climate and ecosystem changes worldwide, especially in developing countries, with approximately 4.2 million premature mortalities according to the World Health Organization (WHO) and the Global Burden of Disease 2015 (GBD2015) [1,2,3]. Particulate matter (PM), especially with an aerodynamic diameter equal to or less than 2.5 (PM_2.5_), has been widely reported for its detrimental consequences for several human body systems, causing multiple diseases, including cancers, cardiovascular diseases, and neurodegenerative diseases such as Alzheimer’s disease, Parkinson’s disease, ischemic stroke, and mental illness [4,5,6,7]. Several previous studies have shown that exposure to PM_2.5_ results in various biological events, including mitochondrial dysfunction, oxidative stress, neuroinflammation, and neuronal death, which ultimately led to neurological disorders [8,9,10].

Polycyclic aromatic hydrocarbons (PAHs), one of the organic substances found in PM_2.5_, are generated via the incomplete combustion of various environmental components such as plants, woods, fossil fuels, and food products [11]. According to the United States Environmental Protection Agency (USEPA), 16 PAHs are categorized based on their levels of toxicity and identified as crucial pollutants, which mostly contaminate food and cause adverse health effects and affect the well-being of humans due to their carcinogenic, mutagenic, or teratogenic toxicities, such as benzo(a) pyrene, naphthalene, and anthracene [12]. Anthracene is one of the solid PAHs comprising three fused benzene rings, which is indicated as a highly significant and non-carcinogenic substance by the US Occupational Health and Safety Administration [13]. Previous studies reported that anthracene induced the generation of intracellular ROS in the freshwater flagellate alga, *Euglena agilis* Carter, and enhanced ROS-mediated phototoxicity in epidermal cells [14,15]. In addition, exposure to anthracene has been previously reported for its neurotoxicity by impairing cholinergic, monoaminergic, and purinergic transmission via inhibiting acetylcholinesterase (AChE) and monoamine oxidase (MAO) and promoting oxidative stress-induced neuronal damage and death in neuronal cell lines [16]. However, the neuroinflammatory effects of anthracene on glial cells have not been fully elucidated. 

Microglia, the resident immune cells, are responsible for the regulation of the immune system in the brain and mainly contribute to neuroinflammatory responses. The activation of microglia carries both beneficial and detrimental roles in inflammatory induction [17]. The uncontrolled and persistent activation of immune cells in the brain leads to chronic inflammation, called neuroinflammation, which ultimately results in the excessive production of inflammatory-related substances such as pro-inflammatory cytokines, including tumor necrosis factor-α (TNF-α), interleukin 1β (IL-1β) and interleukin 6 (IL-6), and reactive oxygen species (ROS) [18]. These consequences result from the dysregulation of various intracellular signaling cascades including MAPKs, such as the c-Jun *N*-terminal kinases (JNKs), the extracellular signal-regulated kinases (ERKs) and the p38MAPKs, and the toll like receptor/nuclear factor kappa-light-chain-enhancer of activated B cells (TLR/NF-κB) [19,20]. Hence, there has been extensive research into inhibiting microglia-mediated neuroinflammation in the central nervous system (CNS) to alleviate neuroinflammatory-related disorders induced by PAHs.

In general, non-steroidal anti-inflammatory drugs (NSAIDs) are widely applied for attenuating inflammatory-related disorders through the selective suppression of COX activity. However, harmful effects due to long-term usage have been identified in various organs such as the gastrointestinal, renal, and cardiovascular systems [21]. As a result of these adverse consequences, natural plants and herbs and active ingredients have been gaining attention, with attempts to elucidate their pharmacological effects on inflammatory-mediated diseases. *Areca catechu* L. (AC), or areca nut, is a traditional herbal medicine belonging to the Arecaceae family widely spread over the South and Southeast Asia regions. This fruit comprises a husk and kernel, and it is utilized for edible and medical purposes [22]. Previous reports had shown a wide variety of active ingredients within areca nut, including alkaloids such as arecoline, polyphenols, flavonoids, and tannins such as (−)-epicatechins [23]. Its bioactive substances have been determined by its various biological effects, including anti-bacterial activity, and antioxidant and anti-inflammation effects [24,25,26]. However, the anti-neuroinflammatory effects of areca nut and its bioactive components on anthracene-induced BV-2 cells have not been fully investigated. Therefore, in this study, we aimed to evaluate the anti-neuroinflammatory actions and underlying mechanisms of AC extracts, including hexane (ACH), ethyl acetate (ACEA), and ethanol (ACEE) and their active compounds in response to anthracene-mediated neuroinflammatory characterization in BV-2 mouse microglial cells. 

## 2. Materials and Methods

### 2.1. Materials and Reagents

Anthracene (A0495) was purchased from TCI America; arecoline (31593), (−)-epicatechin (E1753), syringic acid (S6881), and resveratrol (purity ≥ 99%) were obtained from Sigma-Aldrich Co. (St. Louis, MO, USA). Dulbecco’s Modified Eagle’s Medium (DMEM) and Bradford reagent were purchased from Sigma-Aldrich Co. (St. Louis, MO, USA). Fetal bovine serum and 0.25% trypsin-EDTA were purchased from Gibco BRL (Life Technologies, Paisley, UK). 3-(4,5-dimethylthiazol-2-yl)-2,5-diphenyltetrazolium bromide (MTT) was obtained from Bio Basic (Markham, ON, Canada). GENEzol reagent was obtained from Invitrogen (Carlsbad, CA, USA). All primers were synthesized and purchased from Bioneer (Daejeon, Republic of Korea). AccuPower RT-premix and 2X GreenStarTM qPCR Master Mix were obtained from Bioneer (Daejeon, Republic of Korea). NE-PER™ Nuclear and Cytoplasmic Extraction Reagents were obtained from Thermo Scientific (Rockford, IL, USA). Antibodies against phosphorylated (p)-JNK (81E11), JNK (9252), p-p38 (D3F9), p38 (D13E1), p65 (D14E12), lamin (D4Q4Z), alpha (α)-tubulin (11H10), and β-actin (13E5) were purchased from Cell Signaling Technology (Danvers, MA, USA), and the antibody against HO-1 (A-3) was obtained from Santa Cruz Biotechnology (Dallas, Texas, USA). The ELISA kit for TNF-α was obtained from Thermo Scientific (Rockford, IL, USA). All solvents, including hexane, ethyl acetate, ethanol, and dimethyl sulfoxide, were purchased from RCI labscan (Bangkok, Thailand).

### 2.2. Preparation of AC Extracts

AC fruits were collected from a local market in Surat Thani in 2022 and were then botanically authenticated by the herbarium of Kasin Suvatabhandhu, Department of Botany, Faculty of Science, Chulalongkorn University, Bangkok, Thailand, where their voucher specimen (BCU 016434) was submitted. Before the extraction process, the husk of the areca nut fruits was separated and then washed, dried, and finely ground into a fine powder. About 40 g of AC powder was extracted with 400 mL of three different polar solvents, namely hexane, ethyl acetate, and ethanol, using a Soxhlet apparatus. Then, the evaporation of all the extracts was carried out using a rotary evaporator at 45 to 50 °C. Finally, the crude extracts were dissolved in DMSO to make a stock solution at 100 mg/mL which was stored at −20 °C in the darkness until further use.

### 2.3. Liquid Chromatography–Mass Spectrometry (LC-MS) Analysis

The phytochemical profiling of bioactive compounds in ACEE was determined by using liquid chromatography coupled with a quadrupole time-of-flight (QTOF) analysis (Bruker Daltonics, Bremen, Germany). An Intensity Solo C18 column with ID 2.1 mm and particle size of 1,8 μm and 100 mm was applied with a mobile phase containing 0.1% ammonium formate buffer in water (A) and 100% methanol (B). A flow rate was set to 0.4 mL/min with a gradient containing 0.1% B for 0–10 min, 25% B for 10–12 min, 80% B for 12–21 min, 90% B for 21–23 min, and 0.1% B for 23–26 min. Next, the electrospray ionization (ESI) 500 V with ion-positive mode was used as a detector, and the *m*/*z* values were analyzed by comparing the KNApSAcK (Keyword Search Web Version 1.000.01) databases and active ingredients in previous reports, with an accepted error of molecular weight less than 30 parts-per-million (ppm).

### 2.4. Molecular Docking

Molecular docking between the candidate ligands and target proteins was performed using AutoDock 4.2.6 software (The Scripps Research Institute, San Diego, CA, USA) with default parameters. The protein and ligand structure, along with the grid boxes, were described in the previous reports [27,28,29,30]. The conformation with the lowest binding energy was selected to evaluate the protein–ligand interaction using BIOVIA Discovery Studio 2020 (San Diego, CA, USA).

### 2.5. Lipinski’s Rule and Pharmacokinetic Property Analysis

To predict the drug-likeliness properties of the possible bioactive compounds, Lipinski’s rule of five parameters was computed by the SwissADME online database (http://www.swissadme.ch) (accessed on 19 May 2024). Additional information related to the pharmacokinetic property of the small molecules was also predicted using SwissADME, along with preADMET (https://preadmet.qsarhub.com/toxicity) (accessed on 19 May 2024) and pkCSM (https://biosig.unimelb.edu.au/pkcsm/) online servers (accessed on 19 May 2024).

### 2.6. Cell Culture and Treatment

The BV-2 mouse microglial cell line (Cat. #ABC-TC212S) was purchased from AcceGen Biotech (Fairfield, NJ, USA) and was maintained in DMEM containing 10% FBS and 1% penicillin/streptomycin under a humidified atmosphere with 5% CO_2_ at 37 °C. After reaching approximately 80% confluence, cells were cultured on different types of cultured plates for 24 h and were treated with anthracene, an inflammatory inducer, in the presence or absence of the extracts and compounds for the indicated doses and time, depending on the experiments. 

### 2.7. Cell Viability Assay

To examine the cytotoxicity of AC extracts, pure compounds including resveratrol, arecoline, (−)-epicatechin, and syringic acid, and anthracene in BV-2 cells, a density of 2.0 × 10^4^ of cells were plated on 96-well culture plate for overnight. Then, the cells were incubated with various concentrations of AC extracts between 0 and 100 μg/mL, pure compounds at 10 μM, and anthracene ranging from 0 to 20 μM for 24 h. Following the incubation, MTT reagent at a dose of 5 mg/mL was added into the well and incubated for another 4 h. After completing the incubation time, the solution was gently removed and replaced by DMSO to dissolve the intracellular purple formazan crystals product, which indicates the viable cells. The reaction was detected using an EnSpire^®^ Multimode Plate Reader (Perkin-Elmer, Waltham, MA, USA) by measuring the optical density at a wavelength of 570 nm. The results are displayed as a percentage relative to the untreated group.

### 2.8. RNA Extraction and qPCR Analysis

Total RNA was isolated from BV-2 cells at a density of 4.0 × 10^5^, which were treated with AC extracts or compounds in the presence of anthracene for 3 h using GENEzol solution (Invitrogen), according to the manufacturer’s protocol. Then, 1 μg/mL of isolated RNA was reverse-transcribed into it, and then the relative expression levels of the target gene were measured by conducting a qPCR analysis, starting with pre-denaturation at 95 °C for 10 min, denaturation at 95 °C for 20 s, annealing at 58 °C for 40 s, and a melting curve analysis. All primer sequences are shown in Table 1. The 2^−ΔΔCt^ method was applied to calculate the relative expression of the target genes, which was normalized with β-actin.

### 2.9. Enzyme-Linked Immunosorbent Assay (ELISA)

After the treatment of cells with ACEE or compounds together with anthracene for 24 h, the cultured medium of all the samples was centrifuged to remove cell debris, and the cell supernatant was further collected and kept at −80 °C. The level of TNF-α cytokine was measured by using the commercial kit TNF-α ELISA according to the manufacturer’s instruction. The optical density was also determined at a wavelength of 450 nm. 

### 2.10. Intracellular ROS Analysis

The BV-2 cells at a density of 2.0 × 10^4^ of cells/well were plated on a 96-well culture plate for overnight, followed by treating the ACEE or compounds with anthracene for 24 h. Then, cells were gently washed with 1× PBS and were treated with H_2_DCFDA at a working dose 10 μM for 45 min, followed by washing with PBS. The fluorescent intensity was detected at a wavelength of excitation and emission of 485 and 535, and the fluorescent images were visualized under the Cytation7 Cell Imaging Multimode Reader (BioTek, Winooski, VT, USA). Hydrogen peroxide (H_2_O_2_) was used as a positive control of ROS stimulation. 

### 2.11. Immunofluorescence Analysis

BV-2 cells were plated on a 12-well culture plate containing round coverslips and then were incubated with ACEE or compounds in the presence of anthracene for 1 h. After treatment, all coverslips were subsequently transferred to a new plate to perform immunostaining. Briefly, the coverslips were washed 3 times with 1× PBS and were further fixed and permeabilized with PBS containing 4% paraformaldehyde and 0.25% Triton X-100, respectively, for 20 min. After that, 5% BSA was added to block any non-specific proteins for another 1 h, followed by incubating the primary antibody at 4 °C for overnight. Next, cells were rinsed with PBS before incubating with the secondary antibody at room temperature for 45 min, followed by DAPI for 10 min. Finally, samples were washed and mounted on a glass slide using a mounting reagent. All fluorescent images were captured on the LSM980 confocal laser scanning microscope with airyscan 2 (CLSM, ZEISS, Jena, Germany) at the Oral Biology Research Center (ORAC), Faculty of Dentistry, Chulalongkorn University. 

### 2.12. Preparation of Cytoplasmic and Nuclear Proteins

BV-2 cells were seeded on 60 mm culture dishes at a density of 2.0 × 10^6^ and were subsequently treated with ACEE or compounds together with anthracene for 1 h. After exposure time, cytoplasmic and nuclear proteins were separated using NE-PER™ Nuclear and Cytoplasmic Extraction Reagents according to the manufacturer’s protocol. These proteins were kept at −80 °C until use. 

### 2.13. Whole Protein Extraction and Immunoblotting Analysis

BV-2 cells were plated on a six-well culture plate and further incubated with ACEE or compounds in the presence or absence of anthracene for specific time. Then, whole proteins were collected using cold 1× RIPA buffer consisting of proteinase inhibitor and phenylmethylsulfonyl fluoride (PMSF) and centrifuged at 12,000 rpm for 10 min at 4 °C. The concentration of all cell lysates, including cytoplasmic, nuclear, and total proteins, was compared with bovine serum albumin (BSA) using Bradford reagent. An equal concentration of all samples was separated by sodium dodecyl sulfate–polyacrylamide gel and further transferred to a polyvinylidene difluoride (PVDF) membrane. After that, 5% skim milk or BSA, diluted in Tris-buffered saline and 0.1% Tween 20 (TBS-T), was used to block any specific proteins for 1 h, and then all membranes were probed with primary antibodies, including p65 (1:1000), p-p38 (1:1000), p38 (1:2000), p-JNK (1:1000), JNK (1:2000), HO-1 (1:2000), lamin (1:2000), α-tubulin (1:2000), and β-actin (1:2000) at 4 °C overnight. Next, all membranes were incubated with horseradish peroxidase (HRP)-conjugated secondary antibody (1:8000) for 45 min. Finally, the visualization of target proteins was carried out using an enhanced chemiluminescence (CL) reagent with Amersham^TM^ ImageQuant 800 imaging system (Cytiva, Marlborough, MA, USA), and then they were quantified using ImageJ software version 1.53k (NIH, Bethesda MD, USA).

### 2.14. Statistical Analysis

All experiments were conducted at least in triplicate independently, and the results are displayed as the means ± standard variation. The statistical significance was considered as the *p*-value less than 0.05 by performing a one-way ANOVA with Tukey’s multiple comparison analysis with SPSS statistics version 29.0.1 software.

## 3. Results

### 3.1. Characterization of Phytochemical Components in the ACEE

**Figure 1:**
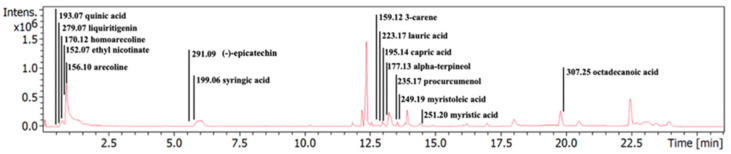
LC-MS chromatogram for ACEE. The graph depicts the candidate bioactive constituents in ACEE that could possess a vital role in the anti-neuroinflammation.

The phytochemical profiling of the ACEE was examined using LC-MS analysis. Table 2 shows how various bioactive components, including quinic acid, liquiritigenin, homoarecoline, ethyl nicotinate, arecoline, (−)-epicatechin, syringic acid, 3-carene, lauric acid, capric acid, alpha-terpineol, procurcumenol, myristoleic acid, myristic acid, and octadecanoic acid were proposed in ACEE by comparing the KNApSAcK database and previous reports. Additionally, the chromatographic peaks of ACEE in ion-positive mode are shown in Figure 1.

### 3.2. In Silico Evaluation of Identified Compounds in ACEE and NF-κB Protein

**Figure 2:**
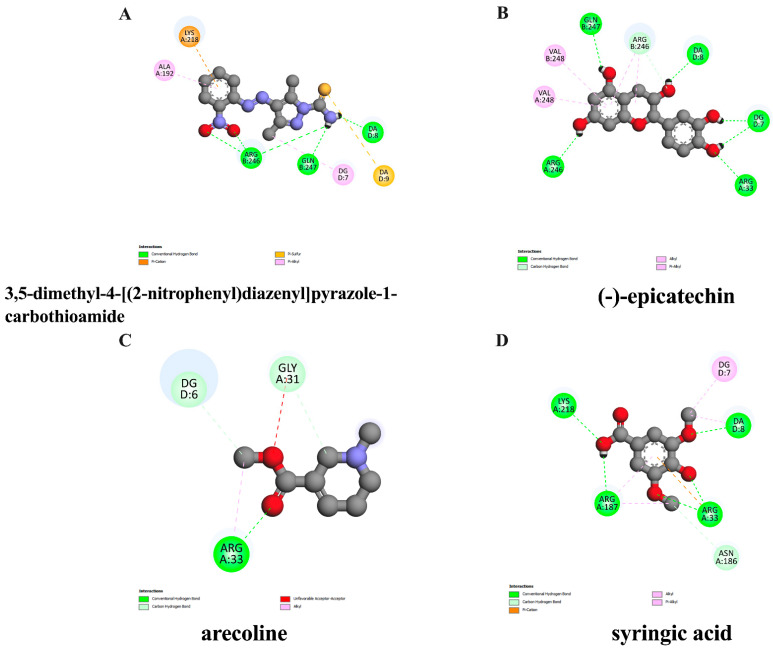
Schematics of interactions of amino acid residues between native ligand or identified compounds and NF-κB protein. The original suppressor of NF-κB protein is (**A**) 3,5-dimethyl-4-[(2-nitrophenyl)diazenyl]pyrazole-1-carbothioamide, and the candidate substances are as follows: (**B**) (−)-epicatechin, (**C**) arecoline, and (**D**) syringic acid. The green dashed line represents hydrogen, the pink or purple dashed lines represent hydrophobic bonds, and the yellow dashed line indicates other bonds.

To screen whether the candidate pure compounds possibly found in ACEE exert suppressive effects on anthracene-induced inflammation, the interaction between inflammatory-related proteins such as NF-κB and selected compounds found in ACEE, including arecoline, (−)-epicatechin, and syringic acid, was determined using molecular docking analysis. The results showed that the binding affinity of the native inhibitor, 3,5-dimethyl-4-[(2-nitrophenyl) diazenyl] pyra-zole-1-carbothioamide, was −6.47 kcal/mol, and (−)-epicatechin exerted the closest binding energy at −6.08 kcal/mol compared to the native ligand as shown in Table 3, and the interaction between ligands and protein is demonstrated in Figure 2. Although (−)-epicatechin showed the lowest binding energy with NF-κB, all selected compounds, including arecoline, (−)-epicatechin, and syringic acid, were also explored for their inhibitory effects in a cell-based assay. 

### 3.3. Lipinski’s Rule of Five Parameters and ADMET Properties of Pure Compounds in ACEE

To evaluate the drug-likeliness properties of the bioactive compounds, Lipinski’s rule of five parameters analysis was carried out. The rules of Lipinski include a molecular weight lower than 500 Da, hydrogen bond acceptors lower than ten, hydrogen bond donors lower than five, and a MlogP lower than 4.15, and any compounds which break the rules of under one parameter are acceptable as having drug properties. The results showed that all active substances passed the criteria of Lipinski’s regulation as shown in Table 4. Moreover, these compounds were also analyzed through ADMET analysis, which is associated with the evaluation of pharmacokinetics and pharmacodynamic properties, namely the absorption, distribution, metabolism, excretion, and toxicity, whose results are shown in Table 4. In addition, as shown in Table 5, all three compounds showed a high ability to be absorbed via the gastrointestinal tract. However, some compounds, especially arecoline and syringic acid, showed a carcinogenic effect on mice and rats. Moreover, these compounds showed differences in their ability for BBB penetration, which was indicated by a log BB with a cut-off as follows: log BB values equal to or more than 0.3 suggest that the compound can readily pass through the BBB, those with log BB values less than 0.3 and more than −1 could still pass through the BBB, and those with log BB values less than −1 are unable to pass through the BBB [31]. The result showed that arecoline was the highest capable bioactive to penetrate and pass through the BBB. 

### 3.4. Effect of ACEE and Pure Compounds on the Viability of BV-2 Cells

**Figure 3:**
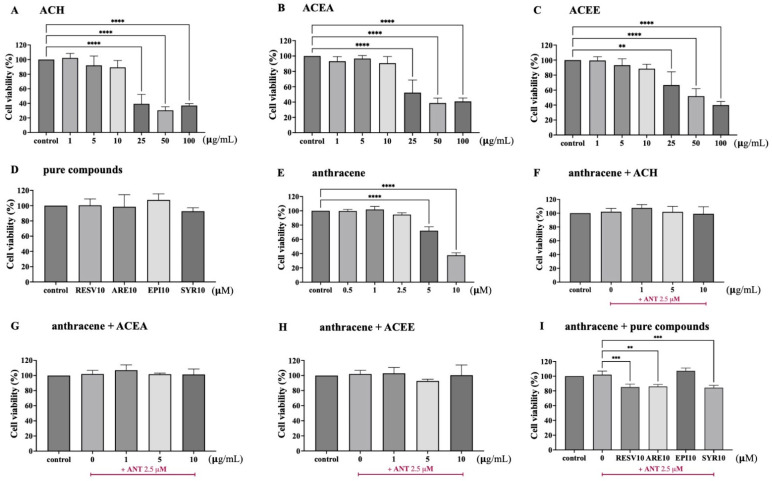
Cytotoxicity effects of AC extracts, pure compounds, and anthracene on BV-2 cells. Cell viability was assessed using an MTT assay. BV-2 cells were treated with AC extracts, including (**A**) ACH, (**B**) ACEA, (**C**) ACEE, or (**D**) pure compounds, including resveratrol (RESV), arecoline (ARE), (−)-epicatechin (EPI), and syringic acid (SYR), and (**E**) anthracene for 24 h. Additionally, the cells were co-treated with anthracene and (**F**) ACH, (**G**) ACEA, (**H**) ACEE, or (**I**) compounds for 24 h. Data are represented as the mean ± SD from at least three independent experiments. A *p*-value < 0.05 was considered as a significant difference between each group. (**** *p* < 0.001, *** *p* < 0.001, ** *p* < 0.01 vs. control (DMSO-treated) or anthracene-treated group).

The in vitro assessment of the cell viability of BV-2 cells upon treatment with various doses of AC extracts (0–100 μg/mL), pure compounds including resveratrol, arecoline, (−)-epicatechin, and syringic acid (10 μM), and anthracene (0–10 μM) for 24 h was primarily performed by MTT assay and is illustrated in Figure 3A–C, D, and E respectively. The results showed that treatment alone with more than 25 μg/mL of AC extracts, including hexane (ACH), ethyl acetate (ACEA), and ethanol (ACEE), significantly decreased the viable cells, compared with the DMSO control, whereas 10 μM of pure compounds, including resveratrol, arecoline, (−)-epicatechin, and syringic acid, had no significant effect on cell viability as shown in Figure 3A–D. Additionally, to elicit an inflammatory response in BV-2 cells, anthracene was selected as an inflammatory inducer. The MTT assay results demonstrated that the anthracene-exposed cells increased cytotoxicity at a concentration more than 5 μM (Figure 3E). Moreover, the co-treatment of the selected doses exhibited a more than 80% cell survival of the extracts or compounds, whilst anthracene revealed no cytotoxic effects on BV-2 cells (Figure 3F–I). Therefore, AC extracts at a concentration lower than 25 μg/mL, pure compound at 10 μM, and anthracene at 2.5 μM were considered for further experiments.

### 3.5. Inhibitory Effect of ACEE and Pure Compounds on the Levels of Pro-Inflammatory Cytokines

**Figure 4:**
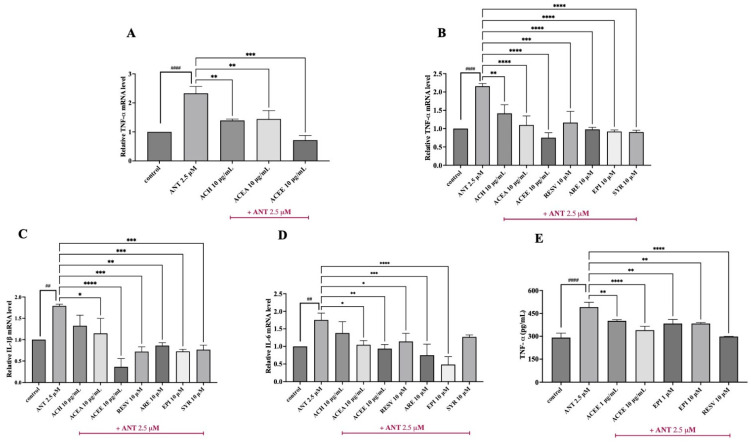
Effects of ACEE and pure compounds on the mRNA and protein levels of pro-inflammatory cytokines in BV-2 cells. The mRNA expressions of inflammatory mediators were examined after the treatment of cells with ACEE or pure compounds, namely resveratrol (RESV), arecoline (ARE), (−)-epicatechin (EPI), and syringic acid (SYR), in response to anthracene for 3 h using qPCR. The relative expression, as shown by fold changes in (**A**,**B**) TNF-α, (**C**) IL-1β, and (**D**) IL-6, were normalized with the internal control, β-actin. The release of (**E**) TNF-α was also measured using a commercial ELISA kit after co-treatment for 24 h. The release of TNF-α (pg/mL) in the cell culture medium was calculated by comparison to the standard curve. Data are represented as the mean ± SD from at least three independent experiments. A *p*-value < 0.05 was considered as a significant difference between each group. (#### *p* < 0.0001, ## *p* < 0.01 vs. control (DMSO-treated), **** *p* < 0.0001, *** *p* < 0.001, ** *p* < 0.01, * *p* < 0.05 vs. anthracene-treated group).

To investigate whether the AC extracts manifested anti-neuroinflammatory effects against anthracene-stimulated BV-2 cells, the mRNA levels of three main pro-inflammatory mediators, including TNF-α, IL-1β, and IL-6 were, measured using qPCR analysis. After the co-treatment of cells with AC extracts in the presence or absence of anthracene for 3 h, a significant increase in all tested cytokines was represented, as compared with control. Among three fractions of AC at 10 μg/mL, ACEE showed the highest inhibitory effects on TNF-α expression (Figure 4A), compared to the anthracene group. qPCR results also showed that ACEE could suppress the expression of all cytokines in a dose-dependent manner compared to anthracene treatment, as shown in Figure 4B–D. Thus, ACEE and its identified compounds, including arecoline, (−)-epicatechin, and syringic acid, were selected for further analysis. Interestingly, we found that the mRNA levels of these cytokines were also reduced after treatment with all three compounds (Figure 4B–D). Resveratrol, which was used as a positive inhibitor, showed similar inhibitory effects as found in ACEE and compounds. Among three compounds identified in ACEE, (−)-epicatechin showed the most promising effects on suppressing the mRNA expression levels of pro-inflammatory cytokines TNF-α, IL-1β, and IL-6. This corresponds to the results from the docking analysis that (−)-epicatechin exhibited the lowest binding energy for the key regulator of inflammation, NF-κB. Thus, (−)-epicatechin was selected for study along with ACEE in the rest of the experiments. Next, the release of cytokines such as TNF-α was examined using a commercial ELISA kit, and the results showed that 24 h of anthracene exposure greatly enhanced the expression of TNF-α in the BV-2 cell culture medium, whereas ACEE and (−)-epicatechin attenuated its expression, as shown in Figure 4E. Therefore, these data suggest that ACEE and (−)-epicatechin imparted anti-neuroinflammatory actions on the mRNA and protein levels of pro-inflammatory cytokines in anthracene-induced BV-2 cells. 

### 3.6. Inhibitory Effect of ACEE and (−)-Epicatechin on ROS Production

**Figure 5:**
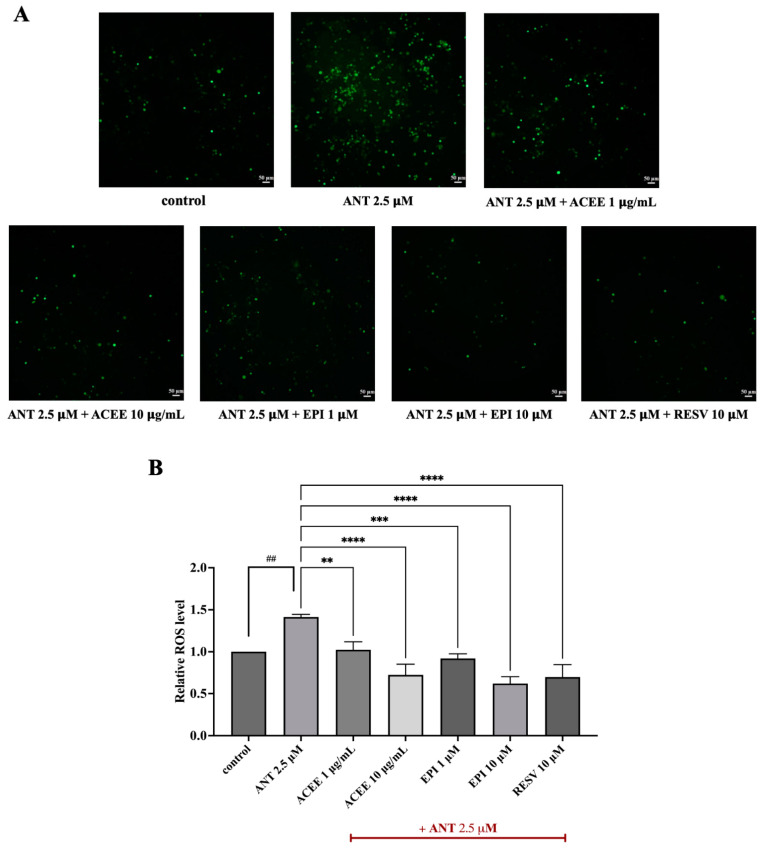
Effects of ACEE and (−)-epicatechin on intracellular ROS production in BV-2 cells. (**A**) The generation of intracellular ROS was visualized under fluorescent microscope using a Cytation 7 machine. Scale bar: 50 μm. (**B**) An H_2_DCFDA assay was conducted, after the co-treatment of cells with ACEE or (−)-epicatechin in the presence of anthracene for 24 h. Data are represented as the mean ± SD from at least three independent experiments. A *p*-value < 0.05 was considered as a significant difference between each group. (## *p* < 0.01 vs. control (DMSO-treated), **** *p* < 0.0001, *** *p* < 0.001, ** *p* < 0.01 vs. anthracene-treated group).

To determine whether ACEE or (−)-epicatechin could suppress the accumulation of ROS in response to anthracene treatment, intracellular ROS production was detected using an H2DCFDA assay. The results showed that, consistent with a powerful oxidizing agent H_2_O_2_ (positive control), anthracene could induce intracellular ROS generation in BV-2 cells, compared to the control group. Conversely, ACEE or (−)-epicatechin significantly reduced the overproduction of ROS upon anthracene stimulation, as shown in Figure 5B. Furthermore, the accumulation of intracellular ROS was also visualized through the detection of fluorescent images using the Cytation7 Cell Imaging Multimode Reader (BioTek). Upon anthracene treatment, BV-2 cells displayed an increase in the fluorescent signal, which was attenuated by ACEE or (−)-epicatechin as shown in Figure 5A. This demonstrated that ACEE and (−)-epicatechin can reduce intracellular ROS due to the activation of anthracene in BV-2 cells.

### 3.7. Effect of ACEE and (−)-Epicatechin on on MAPKs Signaling Activation

**Figure 6:**
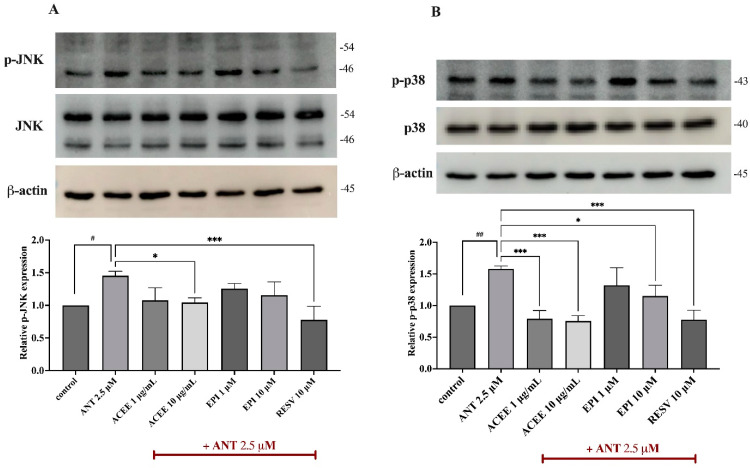
Suppressive effects of ACEE and (−)-epicatechin on MAPKs activation. Cells were co-treated with ACEE or (−)-epicatechin in the presence of anthracene for 15 min, and the relative protein expressions of (**A**) p-JNK and (**B**) p-p38 are represented as histograms, and each protein was quantified and normalized with β-actin. Data are represented as the mean ± SD from at least three independent experiments. A *p*-value < 0.05 was considered as a significant difference between each group. (## *p* < 0.01, # *p* < 0.05 vs. control (DMSO-treated), *** *p* < 0.001, * *p* < 0.05 vs. anthracene-treated group).

To further investigate the underlying pathways which contribute to the anti-neuroinflammatory effects of ACEE and (−)-epicatechin, MAPKs such as JNK and p38, which are the inflammatory signaling cascades, were examined using Western blot analysis. After treating cells with anthracene for 15 min, both p-JNK and p-p38 were significantly upregulated, compared to the control group. However, regarding JNK activation, ACEE at 10 μg/mL only inhibited the phosphorylation of JNK as shown in Figure 6A. In the case of p38 induction, both ACEE and (−)-epicatechin were able to reduce the hyperphosphorylation of p38 in BV-2 cells in response to anthracene as shown in Figure 6B. This suggested that the anti-neuroinflammatory roles of ACEE and (−)-epicatechin are associated with MAPKs signaling activation. 

### 3.8. Effect of ACEE and (−)-Epicatechin on NF-κB Signaling Activation

As NF-κB is a transcriptional protein and a central regulator of inflammatory events, the immunofluorescence and Western blot analysis of p65 were carried out in BV-2 cells which were treated with ACEE or (−)-epicatechin in the presence or absence of anthracene. 

**Figure 7:**
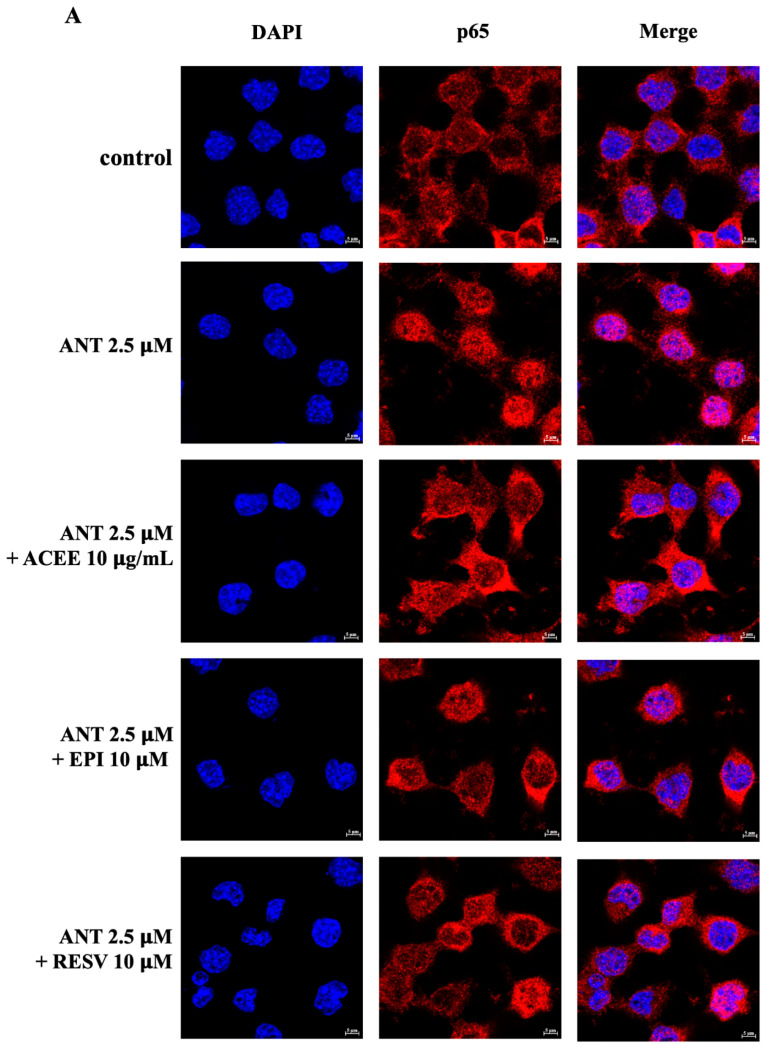

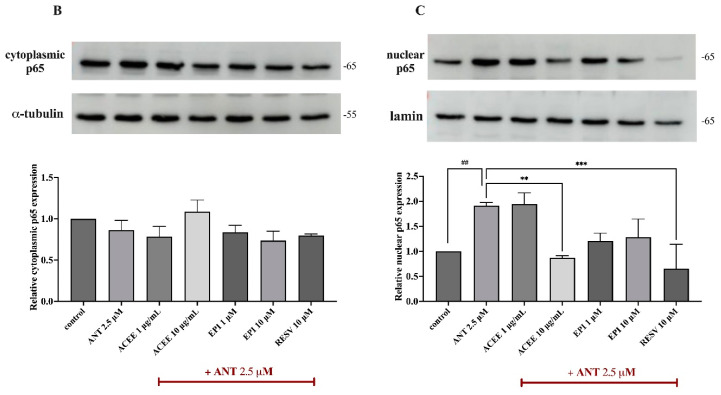
Effects of ACEEE and (−)-epicatechin on NF-κB activation in BV-2 cells. (**A**) BV-2 cells were incubated with ACEE at 25 μg/mL or (−)-epicatechin at 10 μM, in the presence of anthracene 2.5 μM for 1 h to observe the nuclear translocation of p65 using immunofluorescence. The red color from Alexa Flour 555 staining represents p65 in the cells, and the blue color from DAPI staining represents the nucleus. Scale bar: 5 μm. (**B**) The levels of nuclear and (**C**) cytoplasmic p65 were examined by Western blot analysis. The relative expression of nuclear p65 and cytoplasmic p65 was quantified and normalized with lamin and α-tubulin, respectively. Data are represented as the mean ± SD from at least three independent experiments. A *p*-value < 0.05 was considered as a significant difference between each group. (## *p* < 0.01 vs. control (DMSO-treated), *** *p* < 0.001, ** *p* < 0.01 vs. anthracene-treated group).

Upon 1 h of anthracene treatment, the immunofluorescence results showed that BV-2 cells displayed an increase in the nuclear translocation of p65, compared to the control group, whereas ACEE and (−)-epicatechin decreased the red fluorescence level of p65 in the nucleus of BV-2 cells as shown in Figure 7A. To further confirm these changes, the nuclear p65 protein was also determined. Western blot results showed that anthracene greatly enhanced the translocation of p65 into the nucleus of BV-2 cells which were suppressed by ACEE and (−)-epicatechin, whereas cytoplasmic p65 was not significantly changed, as shown in Figure 7B,C. The cumulative data suggested that ACEE and (−)-epicatechin have an anti-neuroinflammatory effect through the regulation of p65. 

### 3.9. Effect of ACEE and (−)-Epicatechin on HO-1 Activation

**Figure 8:**
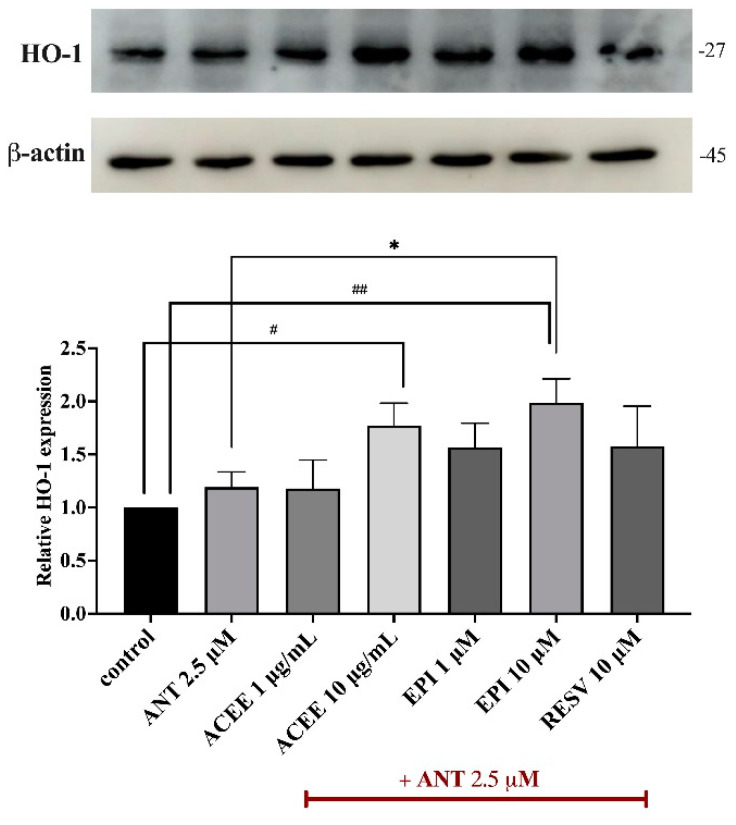
Effects of ACEE and (−)-epicatechin on HO-1 induction. BV-2 cells were incubated with ACEE or (−)-epicatechin in the presence of anthracene for 24 h. The protein expression of HO-1 was measured by Western blot analysis. The histogram graph presents the relative expression of HO-1, which was quantified and normalized with β-actin. Data are represented as the mean ± SD from at least three independent experiments. A *p*-value < 0.05 was considered as a significant difference between each group. (## *p* < 0.01, # *p* < 0.05 vs. control (DMSO-treated), * *p* < 0.05 vs. anthracene-treated group).

To further investigate the related signaling protein which contributed to the inhibitory effects of ACEE and (−)-epicatechin in response to anthracene-induced oxidative stress and neuroinflammation in BV-2 cells, the protein expression of HO-1 was determined using immunoblotting analysis. After co-treatment with ACEE or (−)-epicatechin with anthracene for 24 h, the induction of HO-1 was remarkable, compared to lone treatment with anthracene, as shown in Figure 8.

### 3.10. In Silico Evaluation of Anthracene against Xenobiotic Receptors

**Figure 9:**
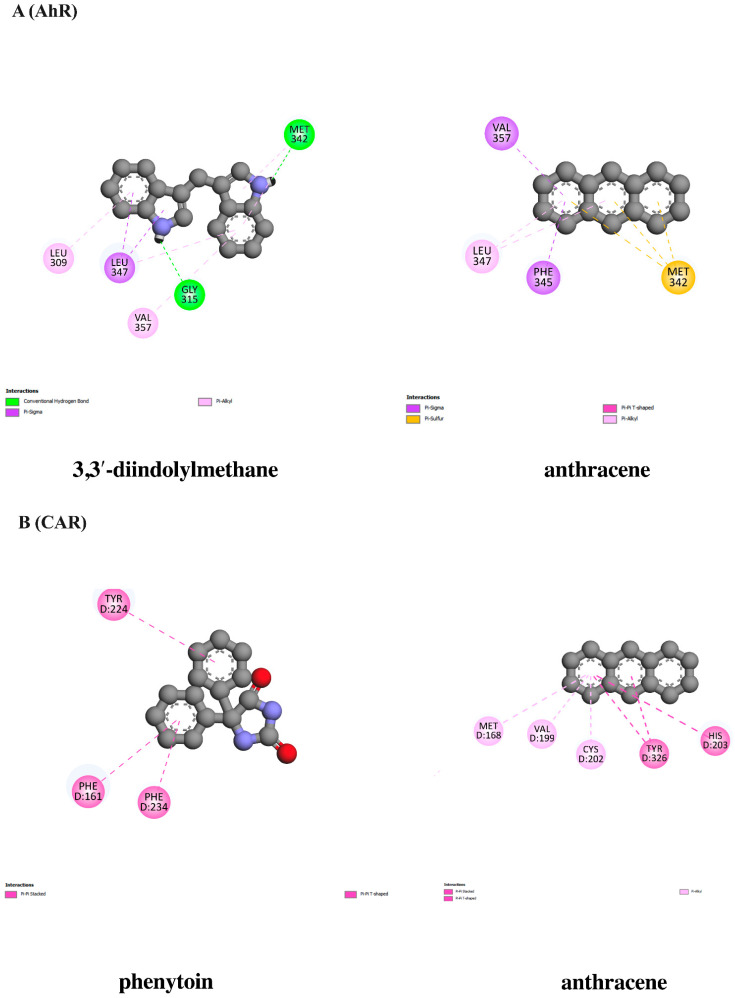

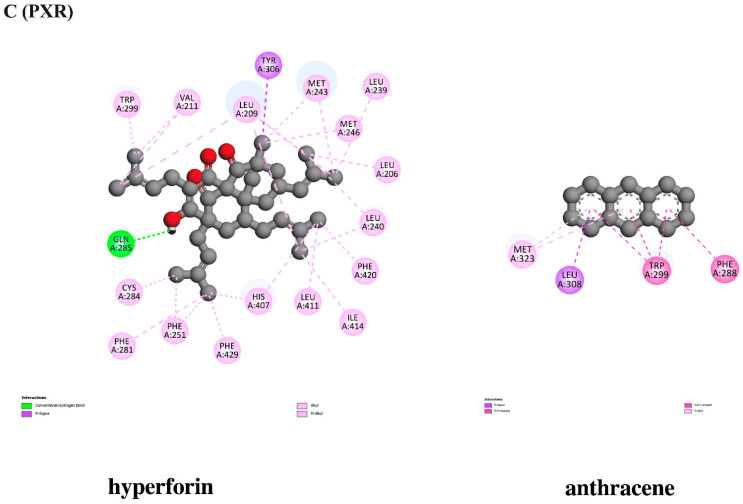
Schematic representation of interactions of amino acid residues between anthracene and xenobiotic receptors. The interactions of anthracene and receptors, including (**A**) AhR, (**B**) CAR, and (**C**) PXR, were compared with individual agonists. which were 3,3′-diindolylmethane, phenytoin, and hyperforin. The green dashed line represents hydrogen, the pink or purple dashed lines represent hydrophobic bonds, and the yellow dashed line indicates other bonds.

To further screen whether anthracene possibly induces inflammatory responses via interacting with xenobiotic receptors, including the aryl hydrocarbon receptor (AhR), the constitutive androstane receptor (CAR), and the pregnane X receptor (PXR), a molecular docking analysis was conducted. The docking results showed that anthracene displayed a binding affinity at −6.82 kcal/mol, which is the closer of AhR agonist, 3,3′-diindolylmethane at −7.71 kcal/mol, and the interaction of anthracene and CAR showed a lower binding affinity at −7.14 kcal/mol compared with the CAR activator, phenytoin. Conversely, anthracene had binding energy at −6.86 kcal/mol, which was higher than the PXR agonist, hyperforin (−10.37 kcal/mol), as shown in Table 6, Table 7 and Table 8. The interaction between ligands and protein is demonstrated in Figure 9. Thus, among the three receptors, anthracene might activate neuroinflammatory signaling cascades partly through CAR. However, further in vitro studies are required. 

## 4. Discussion

Currently, PM_2.5_ causes abnormalities of biological systems in the CNS, including oxidative stress, mitochondrial dysfunction, neuroinflammation, neuronal damage and death, and cognitive deficits which eventually lead to the neurodegeneration [32,33]. Previous studies revealed that PM_2.5_ could distribute throughout the brain, primarily via inhalation through olfactory nerves [34], or enter the lung, causing a large amount of inflammatory mediators to be released into the circulatory system, causing systemic inflammation and resulting in brain damage [35]. Although there are several studies of PM_2.5_-induced neurodegeneration, PAHs, toxicants found in PM_2.5_, should be also more of a focus as to their effects on the CNS.

PAHs are ubiquitous organic toxicants, volatile with lipophilic properties, commonly found in aquatic environments, forests, and the atmosphere, along with contaminating food products and water [36]. The structure of PAHs consists of more than two fused aromatic carbon rings and hydrogens with white or yellow colors. Most PAHs are divided into two groups based on their molecular weight and number of aromatic rings, which are low-molecular-weight (LMW) and high-molecular-weight (HMW) PAHs [36]. 

Additionally, the United States Environmental Protection Agency (USEPA) has established 16 PAHs as significant pollutants, such as benzo(a)pyrene, fluoranthene and anthracene [37]. These PAHs are associated with a wide range of biological consequences, including carcinogenicity, teratogenicity, genotoxicity, and systemic inflammation. Benzo(a)pyrene has been mainly used as a model of PAH-induced toxicity. Previous studies demonstrated its detrimental effects in the CNS by inducing neurotoxicity and cognitive impairment in animal models and humans [38,39,40]. Anthracene, a non-carcinogenic substance containing three fused aromatic benzene rings, is considered as one of the PAHs of high significance and is primarily a contaminant in humans, as it infects the lungs. The 50% lethal doses of anthracene are dependent on the type of the animal model used and exposure routes. For instance, the oral administration of anthracene in mouse and rat showed an LD_50_ at 17,000 mg/kg and 160,000 mg/kg, respectively [13]. In the human epidermal cell line, exposure to anthracene was a photosensitizer inducing acute dermatitis [15]. Recently, anthracene had been investigated for its neurotoxicity, which was induced due to oxidative stress and deteriorated cholinergic function in an HT-22 neuronal cell line [16]. However, the neuroinflammatory effects of anthracene have not been explored.

In order to suppress the neuroinflammation, various NSAIDs are produced. Nevertheless, the prolonged intake of these drugs leads to malicious consequences in the cardiovascular, gastrointestinal tract and renal systems [21]. Hence plant-based bioactive components are used as better replacements to develop immune-friendly anti-inflammatory drugs. Thus, in this study, the anti-neuroinflammatory effects and mechanisms of ACEE and its bioactive compounds were investigated in an anthracene-exposed BV2 mouse microglial cell line. To determine candidate bioactive components in ACEE, an LCMS analysis was carried out. The chromatographic diagrams revealed that ACEE contains quinic acid, liquiritigenin, homoarecoline, ethyl nicotinate, arecoline, (−)-epicatechin, syringic acid, 3-carene, lauric acid, capric acid, alpha-terpineol, procurcumenol, myristoleic acid, myristic acid, and octadecanoic acid as shown in Table 2, which were previously reported in the KNApSAcK database and several studies [23,41]. It is widely known that areca nut consists of 0.3–0.7% of alkaloids, such as arecaine, arecoline, and homoarecoline, which indicated its genetic toxicity, carcinogenic toxicity, cardiotoxicity, and neurotoxicity [42,43,44,45]. Although these alkaloids exerted their toxicities, the previous studies also reported their beneficial biological effects. Arecoline, which is considered the main active compounds in areca nut, could suppress oxidized LDL-induced inflammatory cytokine production [46]. Liquiritigenin, a flavonoid found in areca nut, was reported to have anti-inflammatory property by inhibiting the production of iNOS and inflammatory cytokines via regulating NF-κB activation in macrophages [47]. (−)-epicatechin and syringic acid are polyphenols found in ACEE that exhibit various pharmacological activities, including antioxidant and anti-neuroinflammation effects [48,49,50,51]. Several fatty acids were also found in ACEE, including lauric acid, capric acid, myristic acid, myristoleic acid, and octadecanoic acid. Capric acid and lauric acid exhibited anti-microbial activity and anti-inflammation effects [52]. Myristic acid attenuated skin inflammation by enhancing IL-10 level [53]. 

We then selected some active compounds within ACEE, including arecoline, (−)-epicatechin, and syringic acid, to screen their inhibitory effects on inflammatory-related proteins such as NF-κB by performing a molecular docking analysis. 3,5-dimethyl-4-[(2-nitrophenyl)diazenyl]pyrazole-1-carbothioamide was used as a native suppressor of NF-κB. The docking results showed that (−)-epicatechin revealed the lowest binding affinity compared with other compounds and native ligands as shown in (Table 3), and the interaction of amino acid is illustrated in (Figure 2), indicating possible inhibitory effects on NF-κB protein. Lipinski’s rule of five and ADMET analyses were also conducted to evaluate the drug-likeliness and pharmacokinetic properties of these compounds. All compounds passed the criteria of Lipinski’s rules as shown in (Table 4) and exhibited a high rate of gastrointestinal absorption, with some carcinogenic effects in mouse and rat as found in arecoline and syringic acid, as shown in Table 5. Although the ADMET analysis demonstrated that (−)-epicatechin had the lowest ability of BBB penetration, previous reports showed that (−)-epicatechin could penetrate through the BBB by analyzing microdialysis samples using the HPLC method [54]. Some studies also supported the toxicity of arecoline by reporting its oral-carcinogenic effects on human [44,55] which seriously require consideration. Therefore, based on an in silico study, among all compounds, (−)-epicatechin was taken as the priority component to investigate its anti-neuroinflammatory actions, but other compounds should be also examined for their effects by cell-based approaches. 

In a cell-based assay, we primarily determined the optimal concentrations of the extracts, pure compounds, and anthracene by performing an MTT assay to examine their cytotoxicity in BV-2 cells. The MTT results showed that AC extracts at 0–10 μg/mL, pure compounds including resveratrol, arecoline, (−)-epicatechin, and syringic acid at 10 μM, and anthracene at 2.5 μM exerted a more than 80% cell viability of BV-2 cells, compared to the DMSO control, as shown in Figure 3. A concentration up to 25 μg/mL of all crude extracts greatly reduced cell survival, indicating their toxicity in the mouse cell line. A previous anti-inflammatory study of ethanolic leaf extract of AC also exerted a non-toxic concentration at 10 μg/mL in mouse RAW 264.7 macrophages [56]. To investigate whether AC extracts and compounds inhibit the mRNA expression of inflammatory-related proteins, a qPCR and ELISA assay were performed. Upon the stimulation of inflammatory toxicants, large amounts of pro-inflammatory mediators such as TNF-α, IL-1β and IL-6 were produced and released by glial cells to initiate inflammatory responses [57]. The qPCR results demonstrated that anthracene enhanced the mRNA levels of TNF-α, IL-1β, and IL-6 compared with the control group. Among all the extracts, ACEE exhibited the highest inhibitory effect on TNF-α mRNA expression as shown in (Figure 4A), compared with other extracts at 10 μg/m. So, ACEE was further studied for its suppressive effects on TNF-α, IL-1β, and IL-6 and the results showed that ACEE could significantly reduce the tested cytokines in a dose-dependent manner, compared to anthracene treatment (Figure 4B–D). In addition, pure compounds, including arecoline, (−)-epicatechin, and syringic acid, also demonstrated their inhibitory effects on the expression of these cytokines. 

Based on the in silico analysis and qPCR results, (−)-epicatechin was selected for study in subsequent experiments. Then, the release of TNF-α was measured in anthracene-induced BV-2 cells, and we found that ACEE and (−)-epicatechin attenuated the generation of TNF-α in BV-2 conditioned medium as shown in (Figure 4E). These results are consistent with the previous results that exposure to PAHs could promote the release of TNF-α [58], and areca nut extract could decrease the release of pro-inflammatory cytokines in immune cells [59]. The production of inflammatory cytokines such as TNF-α and IL-6 was mitigated by (−)-epicatechin in response to LPS-mediated macrophages [60]. Furthermore, oxidative stress has been linked to neuroinflammation in neurodegenerative disease progression [61]. To investigate whether ACEE and (−)-epicatechin display antioxidant properties, the production of intracellular ROS was measures using H_2_DCFDA. The result showed that anthracene promoted intracellular ROS generation, whereas ACEE and (−)-epicatechin reversed these changes, as shown in Figure 5A,B. Previous studies reported that anthracene could enhance the level of intracellular ROS in neuronal cells [16], and PAHs also induced oxidative stress and lipid peroxidation [62]. Moreover, AC also exhibited antioxidant and radical-scavenging properties [63], and (−)-epicatechin could suppress oxidative stress in the renal cortex of rat [49]. The overall results suggest that ACEE and (−)-epicatechin possessed antioxidant and anti-neuroinflammatory actions in response to anthracene-exposed BV-2 cells.

Based on the above findings, the underlying signaling cascades involved in the suppression of the anthracene-mediated neuroinflammatory response in BV-2 cells were further elucidated. MAP kinases (MAPKs), including JNK, ERK1/2, and p38, are members of serine/threonine (Ser/Thr) and have been involved in the regulation of inflammatory process by inducing the activation of transcriptional proteins, leading to the production of several inflammatory mediators [64]. The transcription factor NF-κB has been established as a central regulator of the immune system by controlling inflammatory response. The dysregulation of NF-κB has been also implicated in the pathogenesis of neurodegenerative disorders [20,65]. Therefore, the MAPKs/NF-κB signaling pathways have been extensively focused on to explore inflammatory-related mechanisms in neuroinflammatory studies. In this study, the protein expression of p-JNK and p-p38 were evaluated using immunoblotting analysis, and the results showed that the treatment of cells with anthracene promoted p-JNK and p-p38, compared with control. Conversely, the relative expression of p-JNK was inhibited by only ACEE at 10 μg/mL, compared to anthracene treatment (Figure 6A). Interestingly, p-p38 was significantly reduced by both ACEE and (−)-epicatechin (Figure 6B). A previous study showed that areca nut extract could inhibit MAPKs activation in RAW264.7 macrophages [63], and (−)-epicatechin also inhibited inflammatory actions through the MAPKs/NF-κB signaling cascades [66]. In addition, the nuclear translocation of NF-κB was observed under a confocal microscope; the results showed that there was an increase in the nuclear translocation of NF-κB after anthracene stimulation, compared to the control group which was inhibited by ACEE and (−)-epicatechin (Figure 7A). Moreover, the nuclear translocation of NF-κB was also confirmed by Western blot analysis, and the results showed that anthracene promoted the translocation of NF-κB into the nucleus, which was reversed by ACEE at 10 μg/mL. Additionally, (−)-epicatechin tended to reduce nuclear NF-κB as well (Figure 7B,C). There are controversial issues as to whether AC induces or inhibits the inflammation. In the immune cells, areca nut showed anti-inflammatory effects [26], whereas areca nut showed the induction of inflammatory-mediated oral cancer [67,68]. These different effects may be related to types of cells and diseases. Further, an association between HO-1 and transcriptional proteins has been previously reported, including NF-κB, Nrf2, and AP-1, implicating it in antioxidant and neuroinflammation effects [69]. Our study showed that HO-1 was upregulated after the treatment of cells with ACEE at 10 μg/mL and (−)-epicatechin at 10 μM, compared to control. Compared with anthracene treatment, ACEE and (−)-epicatechin tended to increase the induction of HO-1 (Figure 8). Furthermore, previous studies identified the association between PAHs and xenobiotic receptors such as AhR and CAR, and these receptors were also involved in inflammatory processes [70,71,72]. Therefore, a docking analysis was performed to screen whether anthracene enhances the activation of neuroinflammatory-related actions via xenobiotic receptors, including AhR, CAR, and PXR. The results showed that among three receptors, anthracene showed the lower binding energy compared with CAR and the closest binding affinity compared with AhR, as shown in Table 6, Table 7 and Table 8. However, further in vitro studies are required. These obtained data suggested that ACEE and (−)-epicatechin revealed anti-neuroinflammatory effects by reducing inflammatory mediators, including pro-inflammatory cytokines and ROS, by suppressing MAPKs/NF-κB and inducing HO-1 activation. 

## 5. Conclusions

**Figure 10:**
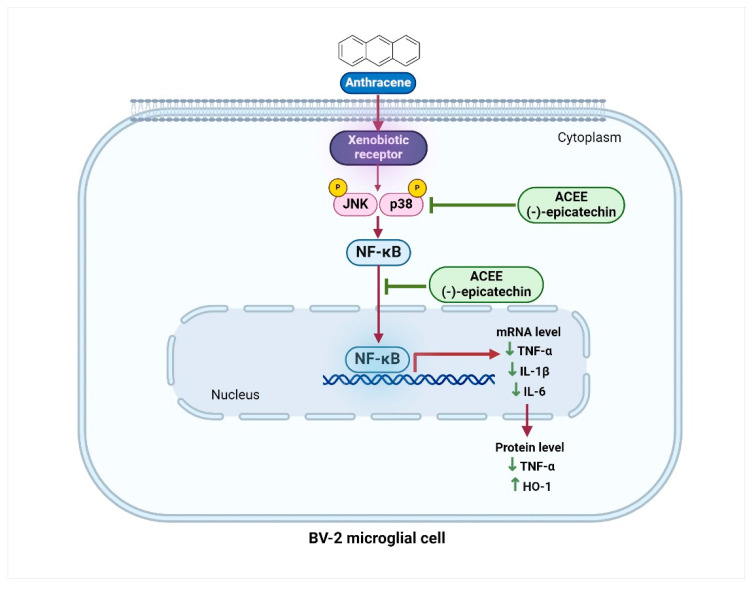
The proposed schematic overview of the anti-neuroinflammatory effects and underlying mechanism of ACEE and (−)-epicatechin in response to anthracene-mediated neuroinflammation in BV-2 mouse microglial cells (created by BioRender.com).

The current study reported for the first time that ACEE and (−)-epicatechin exhibited inhibitory actions on anthracene-induced inflammatory characterization in BV-2 mouse microglial cells, as shown in Figure 10. LC-MS analysis demonstrated certain bioactive constituents within ACEE, such as (−)-epicatechin, arecoline, and syringic acid. The treatment of cells with ACEE or (−)-epicatechin in the presence of anthracene reduced the generation of pro-inflammatory cytokines, including TNF-α, IL-1β, and IL-6. ACEE or (−)-epicatechin also reduced intracellular ROS production in response to anthracene in BV-2 cells. These suppressive effects were regulated by inhibiting MAPKs, such as the JNK and p38 and NF-κB signaling cascades. The induction of HO-1 was also promoted by ACEE or (−)-epicatechin treatment. However, ACEE and its identified components should be more thoroughly elucidated in other glial cells, including primary cells, animal models, and clinical trials. Although the international Agency for Research on Cancer (IARC) indicated the areca nut as a class I carcinogenic substance due to its main bioactive component, arecoline, there are other pure compounds within the areca nut that may provide beneficial effects on the suppression of neuroinflammation without any severe adverse effects. Thus, our current study suggests the potential anti-neuroinflammatory properties of ACEE, which can be further developed as anti-neuroinflammatory agents for the treatment of air pollution-induced neuroinflammation in the CNS.

## Figures and Tables

**Table 1:** All primer sequences used in qPCR.

Gene	Forward Primer (5′ → 3′)	Reverse Primer (5′ → 3′)
IL-1β	GAAATGCCACCTTTTGACAGTG	CTGGATGCTCTCATCAGGACA
TNF-α	GATCGGTCCCCAAAGGGATG	TAGCAAATCGGCTGACGGTG
IL-6	TCTTGGGACTGATGCTGGTG	CAGGTCTGTTGGGAGTGGTA
β-actin	GGCTGTATTCCCCTCCATCG	CCAGTTGGTAACAATGCCATGT

**Table 2:** Proposed bioactive constituents in ACEE.

NO.	Compound Name	Molecular Formula	Retention Time (min)	*m*/*z*	ppm
1.	quinic acid	C_7_H_12_O_6_	0.68	193.0707 [M+H]^+^	2.55
2.	liquiritigenin	C_15_H_12_O_4_	0.73	279.0707 [M+Na]^+^	−28.63
3.	homoarecoline	C_9_H_15_NO_2_	0.85	170.1179 [M+H]^+^	1.06
4.	ethyl nicotinate	C_8_H_9_NO_2_	0.89	152.0700 [M+H]^+^	7.47
5.	arecoline	C_8_H_13_NO_2_	0.9	156.1021 [M+H]^+^	1.92
6.	(−)-epicatechin	C_15_H_14_O_6_	6.16	291.0892 [M+H]^+^	−8.34
7.	syringic acid	C_9_H_10_O_5_	6.47	199.0583 [M+H]^+^	11.62
8.	3-carene	C_10_H_16_	12.66	159.1182 [M+Na]^+^	−23.75
9.	lauric acid	C_12_H_24_O_2_	12.95	223.1702 [M+Na]^+^	−14
10.	capric acid (decanoic acid)	C_10_H_20_O_2_	13.19	195.1394 [M+Na]^+^	−19.19
11.	alpha-terpineol	C_10_H_18_O	13.2	177.1294 [M+Na]^+^	−25.10
12.	procurcumenol	C_15_H_22_O_2_	13.58	235.1701 [M+H]^+^	−1.37
13.	myristoleic acid	C_14_H_26_O_2_	13.63	249.1869 [M+Na]^+^	−17.04
14.	myristic acid (tetradecanoic acid)	C_14_H_28_O_2_	14.57	251.1970 [M+Na]^+^	0.88
15.	octadecanoic acid	C_18_H_36_O_2_	19.94	307.2481 [M+Na]^+^	−11.27

**Table 3:** Molecular docking of identified compounds to NF-κB binding site.

Ligand	Binding Energy (kcal/mol)	Inhibition Constant (μM)	Amino Acid Interaction		
			Hydrogen Bond	Hydrophobic Bond	Other
3,5-dimethyl-4- [(2nitrophenyl)diazenyl]pyrazole-1- carbothioamide (native inhibitor)	−6.47	18.12	ARG246(3) GLN247 DA8	ALA192 LYS218 DG7	LYS218 DA9
(−)-epicatechin	−6.08	34.75	ARG33 ARG246(2) GLN247	GLN263	ASP382(2)
arecoline	−4.84	281.76	ARG33(2) GLY31	ARG33	-
syringic acid	−4.11	970.76	ARG33(2) LYS218 ARG187 ASN186	ARG187(2)	ARG33

**Table 4:** Prediction of drug-likeness of candidate ligands by Lipinski’s rule of five parameters.

Compound	Molecular Weight (≤500)	#H-Bond Acceptors (≤10)	#H-Bond Donors (≤5)	MLOGP (≤4.15)	Lipinski #Violations (≤1)
Arecoline	155.19	3	0	0.58	0
(−)-Epicatechin	290.27	6	5	0.24	0
Syringic acid	198.17	5	2	0.49	0

**Table 5:** ADMET-predicted properties of possible ligands in ACEE.

Pharmacokinetic Property	Arecoline	(−)-Epicatechin	Syringic Acid
GI absorption	High	High	High
Pgp substrate	No	No	No
log Kp (skin permeation) (cm/s)	−7.00	−7.82	−6.77
BBB permeant (log BB)	0.033	−1.054	−0.191
CYP1A2 inhibitor	No	No	No
CYP2C19 inhibitor	No	No	No
CYP2C9 inhibitor	No	No	No
CYP2D6 inhibitor	No	No	No
CYP3A4 inhibitor	No	Yes	No
Carcinogenicity (Mouse)	Positive	Negative	Negative
Carcinogenicity (Rat)	Negative	Negative	Positive
Hepatotoxicity	Yes	No	No
AMES toxicity	No	No	No
hERG inhibition	Low risk	Medium risk	Low risk

**Table 6:** Molecular docking of anthracene to AhR.

Ligand	Binding Energy (kcal/mol)	Inhibition Constant (μM)	Amino Acid Interaction		
			Hydrogen Bond	Hydrophobic Bond	Other
3,3-Diindolylmethane (AhR agonist)	−7.71	2.24	MET342 GLY315	LEU347(3) MET342(2) VAL357 LEU309	-
Anthracene	−6.82	10.09	-	PHE345(2) VAL357 LEU347(2)	MET342(3)

**Table 7:** Molecular docking of anthracene to CAR.

Ligand	Binding Energy (kcal/mol)	Inhibition Constant (μM)	Amino Acid Interaction		
			Hydrogen Bond	Hydrophobic Bond	Other
Phenytoin (CAR agonist)	−6.99	7.56	-	TYR224 PHE234 PHE161	-
Anthracene	−7.14	5.84	-	TYR326(2) HIS203 MET168 VAL199 CYS202	-

**Table 8:** Molecular docking of anthracene to PXR.

Ligand	Binding Energy (kcal/mol)	Inhibition Constant (μM)	Amino Acid Interaction		
			Hydrogen Bond	Hydrophobic Bond	Other
Hyperforin (PXR agonist)	−10.37	0.025	GLN285	TYR306 MET243(2) MET246 CYS284 LEU209(4) LEU239 LEU206 LEU240(2) LEU411 ILE414 VAL211(2) PHE251(2) PHE281 TRP299 HIS407(2) PHE420 PHE429	-
Anthracene	−6.86	9.41	-	LEU308 TRP299(5) PHE288 VAL199 MET323(2)	-

## Data Availability

The data generated and/or analyzed during this study are included in this published article. Other data supporting the findings of this study are available from the corresponding author upon request.
